# Performance of Reverse Transcription Loop-Mediated Isothermal Amplification (RT-LAMP) Targeting the RNA Polymerase Gene for the Direct Detection of SARS-CoV2 in Nasopharyngeal Swabs

**DOI:** 10.3390/ijms241713056

**Published:** 2023-08-22

**Authors:** Elias da Rosa Hoffmann, Lisiane da Rocha Balzan, Everton Inamine, Lisiane Rech Pancotto, Guilherme Gaboardi, Vlademir Vicente Cantarelli

**Affiliations:** 1 Basic Health Sciences Department, Federal University of Health Sciences of Porto Alegre (UFCSPA), Porto Alegre 90050-170, Brazil; hoffmann.elias@gmail.com; 2 Bom Pastor Laboratory, Molecular Biology Department, Igrejinha 95650-000, Brazil; 3 Diagnósticos da America S.A., Novo Hamburgo 93520-340, Brazil; 4 Central Laboratory, Santa Casa de Misericórdia de Porto Alegre, Porto Alegre 90020-090, Brazil; einamine@gmail.com (E.I.);; 5 Biomedical Sciences Department, Serra Gaúcha University Center (FSG), Caxias do Sul 95020-472, Brazil

**Keywords:** reverse transcriptase-mediated isothermal amplification, LAMP, SARS-CoV-2, coronavirus infections, *RP* gene

## Abstract

In 2020, a global pandemic caused by SARS-CoV-2 was declared. Different institutes proposed diagnostic molecular methods to detect the virus in clinical samples. This study aims to validate and standardize the use of a loop-mediated isothermal amplification (LAMP)-based methodology targeting the viral *RP* gene, as a faster and low-cost diagnostic method for SARS-CoV-2 infections. The results obtained with RT-LAMP (Reverse Transcriptase) were compared to the results of real-time polymerase chain reaction (RT-PCR) to assess its sensitivity and specificity. In total, 115 samples (nasopharyngeal samples) were used for detecting SARS-CoV-2 by RT-LAMP, with 43 positives and 72 negatives. The study showed a positive predictive value (PPV) of 90.7% and a negative predictive value (VPN) of 100%. The LAMP assay also demonstrated a high sensitivity of 90.7% and a specificity of 100% (confidence interval 77.9–97.4%) when using the lower detection limit of 40 copies/µL. The RT-LAMP described here has the potential to detect even the new variants of SARS-CoV-2, suggesting that it may not be significantly affected by gene mutations. The RT-LAMP targeting the *RP* viral region is faster and less expensive than other molecular approaches, making it an alternative for developing countries.

## 1. Introduction

At the beginning of the SARS-CoV-2 pandemic, the virus was initially imported into Brazil from Europe, where it quickly found a vulnerable environment due to the lack of proper protective measures. This allowed the virus to spread rapidly within the country, resulting in a devastating outcome. Consequently, multiple variants of concern, such as the P.1 variant, emerged and contributed to the already alarming death toll, which currently stands at over 687 thousand people [1]. The number of COVID-19 cases in Brazil has reached a staggering 35 million to date.

To combat the widespread dissemination of the virus, various measures were suggested, including social isolation and other containment protocols. However, as reports from countries across the globe started pouring in, it became evident that the viral spread and severe respiratory diseases were not limited to Brazil alone. As a result, the total number of COVID-19 cases worldwide has climbed to a startling 657 million, with an alarming death toll of 6.9 million (as of May 2023).

Numerous diagnostic methods have been proposed by different institutions in various countries, each striving to tackle the challenges posed by SARS-CoV-2. The Centers for Disease Control and Prevention (CDC) in the United States, among others, initially advocated for molecular methods such as PCR-based assays, which were later followed by immunological assays as the pandemic progressed [2].

Among the diagnostic techniques, reverse transcription real-time polymerase chain reaction (RT-qPCR) emerged as the primary method for detecting SARS-CoV-2 due to its exceptional sensitivity and ability to identify even minute amounts of viral genetic material in clinical samples. Nonetheless, RT-qPCR does have its drawbacks. It is a time-consuming and expensive technique, making its implementation challenging in certain diagnostic centers, particularly those in low- and mid-income countries.

In an effort to address these limitations, an alternative method called loop-mediated isothermal amplification (LAMP) gained attention. LAMP is an isothermal amplification technique developed by Notomi and colleagues in 2000 [3]. One of its key advantages is the ability to generate billions of DNA copies at a constant temperature, eliminating the need for complex thermocyclers and significantly reducing the overall cost of the test. Additionally, LAMP demonstrates high specificity by targeting up to eight distinct regions of the DNA, making it a reliable and accurate diagnostic method. Furthermore, LAMP can be combined with a reverse transcription step, allowing it to be applied to RNA-based pathogens such as SARS-CoV-2. This suggests the possibility of using LAMP alongside RT-qPCR to enhance diagnostic capabilities [4].

The objective of this study was to develop a colorimetric loop-mediated isothermal amplification assay preceded by a reverse transcription step (RT-LAMP). The target for amplification in this assay was the SARS-CoV-2 polymerase gene. To evaluate the performance of the proposed method, the sensitivity and specificity, as well as the negative and positive predictive values, were calculated using RT-qPCR as the reference method.

## 2. Results and Discussion

In total, 115 nasopharyngeal samples were used for the detection by RT-LAMP. Among the samples tested, a total of 43 samples were determined to be positive for SARS-CoV-2, while 72 samples yielded negative results. These findings were previously established through the utilization of a validated RT-qPCR method targeting the N1 and N2 viral regions. When comparing the results obtained from the RT-LAMP assay with those obtained from RT-qPCR, a total agreement exceeding 96.5% was observed. Furthermore, the RT-LAMP method exhibited a remarkable specificity of 100% and a sensitivity of 90.7%. These values were calculated based on a limit of detection (LOD) of 40 copies/µL.

**Figure 1:**
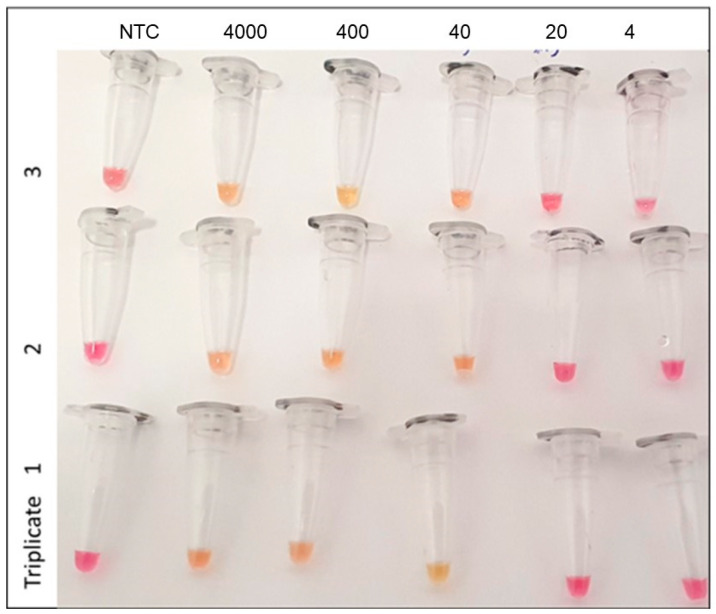
Determination of lower detection limit using RT-LAMP in nasopharyngeal samples. Legend: NTC = No template control. The numbers at the top are copies/µL. Red/pinkish color is a negative reaction. Positive reactions in yellow color.

The negative predictive value (NPV) of the RT-LAMP assay was determined to be 94.7%, indicating a high degree of confidence in identifying true negative cases. Conversely, the positive predictive value (PPV) was found to be 100%, underscoring the reliability of the RT-LAMP assay in accurately detecting positive cases. Notably, the LOD observed for this particular method was 40 RNA copies/µL, which consistently yielded positive results in over 95% of the test runs (as depicted in Figure 1). To ensure robust test validation, these runs were performed in triplicate over 3 days.

It is worth mentioning that the LOD observed with the RT-LAMP assay corresponded to an RT-PCR CT value of less than 27, emphasizing the comparable sensitivity and accuracy of the two diagnostic methods in detecting SARS-CoV-2 infections.

The RT-LAMP assay was employed to analyze a set of positive samples, each with a CT value below 30 in the RT-PCR analysis. These samples encompassed the predominant SARS-CoV-2 variants that were circulating in the specific region under investigation, including the gamma (P.1), delta, and omicron variants. Each variant was represented by five nasopharyngeal samples previously genotyped by a reference laboratory, which were collected in February 2021 and subsequently subjected to the RT-LAMP assay. Notably, the assay successfully and accurately detected all tested variants, implying that the selected primers remained effective even in the presence of potential gene mutations.

This observation provides valuable insight into the robustness and reliability of the RT-LAMP assay, suggesting that the primer sequences employed in the assay were not compromised by the presence of genetic variations within the targeted regions of the SARS-CoV-2 genome. The ability to detect and identify the different variants using the RT-LAMP assay strengthens its applicability as a valuable diagnostic tool capable of accommodating the dynamic nature of the virus. In our setting, the calculated cost of the RT-LAMP is USD 2.5 compared to USD 10 for the current RT-qPCR assay used in our lab.

The RT-LAMP proposed in this study has emerged as a promising alternative to RT-PCR for the detection of SARS-CoV-2 directly from nasopharyngeal swabs, offering numerous advantages and potential applications. One notable advantage is the simplicity of the reaction, which can be performed using a water bath maintained at a fixed temperature of 63 °C. This eliminates the need for expensive equipment, making the RT-LAMP assay a more cost-effective solution compared to RT-PCR, which typically requires specialized thermal cyclers [1].

A significant bottleneck in molecular testing lies in the nucleic acid extraction step, which is not only time-consuming, but also adds to the overall cost of testing. Extraction-free molecular methods, such as the RT-LAMP assay described here, have the potential to significantly reduce the time and costs associated with testing procedures [5]. By bypassing the extraction step, the RT-LAMP assay not only streamlines the workflow, but also minimizes the impact on test sensitivity, further contributing to its cost-effectiveness [6,7].

In the specific setting of this study, the RT-LAMP assay demonstrated robust performance, as indicated by its calculated negative predictive value (NPV) of 100% and a positive predictive value (PPV) of 90.7% (as presented in Table 1. These findings underscore the potential of the RT-LAMP assay as a reliable diagnostic tool for SARS-CoV-2 detection.

Numerous studies investigating the application of RT-LAMP for SARS-CoV-2 detection have consistently reported high sensitivity, with a range of 91% to 100%, even when using different genetic targets and detection strategies [6,7,8,9,10,11,12,13,14,15,16]. While the RT-LAMP assay may occasionally yield false-negative results, especially in cases of extremely low viral loads near the limit of detection (LOD), it is unlikely that such false negatives in this study resulted from *Bst* enzyme inhibition. *Bst* polymerase, the key enzyme used in the RT-LAMP assay, exhibits tolerance to various inhibitors that could potentially impact the performance of RT-PCR assays [17]. It is important to note that low viral loads, particularly during the early or late stages of the symptomatic phase of COVID-19, pose a challenge for all detection methods, including RT-PCR, and may result in missed detections [17].

Recent meta-analyses encompassing large-scale studies involving thousands of SARS-CoV-2-infected patients have revealed that initial false-negative results via RT-PCR can be prevalent, with rates reaching up to 58% [18]. Rapid antigen tests, which have been utilized as an alternative to RT-PCR, also exhibit reduced sensitivity during the earlier stages of COVID-19, with an overall reported sensitivity of approximately 68% [19]. In contrast, the RT-LAMP assay described in this study demonstrates high specificity (100%), which can be attributed to the utilization of six specific primers targeting the selected region, as supported by previous studies [10,13,15,16,20]. This multiple-primer approach enhances the assay’s specificity and further reinforces its reliability for SARS-CoV-2 detection.

According to OPAS [2], one potential drawback of colorimetric LAMP methods, including the RT-LAMP assay, is their susceptibility to acidic samples, such as saliva, which can lead to false-positive results due to the color change of the pH indicator [2]. However, it is crucial to note that in such cases, the appearance of a yellow color upon sample addition to the LAMP mix should prompt the interpretation of the reaction as invalid, mitigating the impact of false positive results. Therefore, as an alternative, turbidity or fluorescence-based tests could be utilized. This emphasizes the importance of following proper protocols and recognizing the limitations of the assay when interpreting the results, particularly in the presence of acidic samples.

In summary, the RT-LAMP assay presented in this study offers a simple, efficient, and cost-effective method for the detection of SARS-CoV-2 in nasopharyngeal samples. By eliminating the need for nucleic acid extraction and utilizing a water bath at a fixed temperature, the assay not only reduces the time and cost associated with testing, but also demonstrates comparable sensitivity to RT-PCR and superior specificity. The ability to complete the assay within 45 min further enhances its appeal, allowing for a rapid and timely detection of the virus [2].

The RT-LAMP assay holds great promise as a valuable tool in resource-limited settings, where access to sophisticated laboratory infrastructure and equipment may be limited. Its user-friendly nature and minimal requirements make it accessible to a broader range of healthcare facilities, facilitating the confirmation of COVID-19 infections in a timely and cost-effective manner.

However, it is essential to acknowledge that the field of SARS-CoV-2 research is continuously evolving, with new viral variants emerging. Further studies are necessary to evaluate the performance of the RT-LAMP assay in the context of these evolving viral genomes. Additionally, expanding the sample size and conducting comparative studies against established diagnostic methods would provide additional validation and strengthen the evidence supporting the efficacy of the RT-LAMP assay for SARS-CoV-2 detection.

In conclusion, the RT-LAMP assay described in this study represents a significant advancement in the field of molecular diagnostics for SARS-CoV-2. Its ability to detect the virus directly from nasopharyngeal swabs, without the need for nucleic acid extraction, coupled with its cost-effectiveness, rapidity, and accuracy, positions it as a valuable alternative to conventional RT-PCR methods. As the global battle against COVID-19 continues, the RT-LAMP assay holds immense potential for widespread implementation, especially in resource-limited settings, contributing to improved patient management and disease control.

## 3. Materials and Methods

### 3.1. Nasopharyngeal Samples

**Figure 2:**
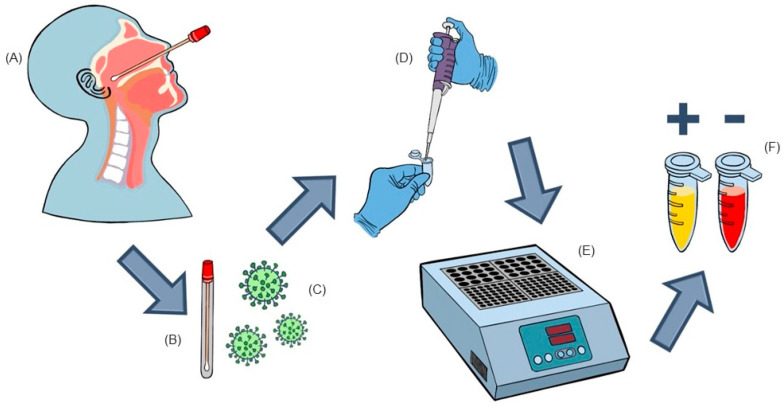
Schematic view of sample collection, transportation, and RT-LAMP analysis. (**A**)—Nasopharyngeal sample collection. (**B**)—Sample is transferred to a tube containing 2 ml of sterile saline. (**C**)—A 1 mL aliquot was transferred to a cryotube and kept frozen until RT-LAMP analysis. (**D**)—Thawed sample added directly to the RT-LAMP mix (New England Biolabs^®^, Ipswich, MA, USA). (**E**)—Tubes were incubated at 63 °C for 45 min. (**F**)—After incubation, the yellow color indicates positivity for SARS-CoV2, while the red color indicates a negative test.

Nasopharyngeal samples employed in this investigation were obtained utilizing a sterile swab and transferred into a tube containing 2 mL of saline solution. The samples were collected at a private laboratory, located in Novo Hamburgo, Brazil, in response to medical requests for RT-qPCR to diagnose COVID-19. The collection period spanned from June 2020 to August 2020, and the samples were promptly frozen and preserved until the time of testing, with a maximum storage time of 24 h. Subsequently, after undergoing RT-qPCR analysis (Promega Corporation, Madison WI, USA) an aliquot of approximately 1 mL of the remaining sample was transferred to a new sterile tube and then stored at a temperature of −20 °C for a maximum of 48 h prior to RT-LAMP testing. It should be noted that during the thawing process, the aliquots were promptly processed to minimize any potential degradation of viral RNA. All samples utilized in this study were handled anonymously and exclusively identified by an internal numerical code, thereby ensuring the confidentiality of patient information, which remained inaccessible and unused throughout the study (as depicted in Figure 2).

In total, the study encompassed a comprehensive set of 115 nasopharyngeal samples. Among these, 43 samples exhibited positive results (with a CT value below 30) for SARS-CoV-2, as determined by RT-qPCR, which served as the benchmark methodology for method comparison [16].

This project was approved by the Ethical Committee of Universidade Federal de Ciências da Saúde de Porto Alegre (UFCSPA-Porto Alegre-Brazil) under the identification number 4,769,584.

### 3.2. Primers for RT-LAMP

Oligonucleotide primers were designed using the online software PrimerExplorer version 5 (https://primerexplorer.jp/e/ (accessed on 22 February 2020)) using the consensus RNA polymerase (*RP*) gene sequence from SARS-CoV-2 as the template. Table 2 shows the chosen primer set designed for this study.

### 3.3. RT-LAMP

The RT-LAMP reaction was carried out utilizing the WarmStart^®^ Colorimetric LAMP Kit, a product manufactured by New England Biolabs^®^ (Ipswich, MA, USA). The laboratory had previously validated the following protocol for the detection of other pathogens, ensuring its reliability and effectiveness. The final reaction volume was set at 10 µL, comprising 5 µL of WarmStart^®^ Colorimetric LAMP MIX, 1 µL of *RP* primers MIX (with a final concentration of 200 nM for F3/B3 primers, 1600 nM for FIP/BIP primers, and 400 nM for LF/LB primers), 2.0 µL of nuclease-free water, and 2 µL of the sample or controls.

Upon preparing the reaction mixtures, the tubes were promptly subjected to an isothermal reaction at a temperature of 63 °C for a duration of 45 min. Following the incubation period, the reactions were visually examined, and a discernible change from the original red/pinkish color to a distinct yellow color was deemed indicative of a positive result. To ensure the validity of the results, positive controls (known positive samples) and non-template controls (NTCs) were included in each new run, serving as reference points for comparison.

It is important to note that for the sample preparation, a brief vortex homogenization step was employed to ensure proper mixing. Subsequently, 2 µL of the homogenized sample was directly used without undergoing any nucleic acid extraction procedures, simplifying the workflow and reducing processing time.

To determine the limit of detection (LOD) for the RT-LAMP assay, a series of 10-fold dilutions were prepared from a pooled positive sample, which had previously been quantified using RT-qPCR. These dilutions were tested in triplicate on multiple occasions. The lowest viral load that consistently produced a clear and repeatedly positive result in the RT-LAMP runs was considered the LOD for this assay. For the concordance analysis, the acceptance criterion was used as a global concordance greater than or equal to 95%.

## Figures and Tables

**Table 1:** Sensitivity and specificity of LAMP compared to RT-PCR.

	LAMP Positive	LAMP Negative
RT-PCR Positive	39	4
RT-PCR Negative	0	72

Sensitivity at LOD 40 copies/µL = 100 × (39/39 + 4) = 90.7% Specificity = 100 × (72/0 + 72) = 100%.

**Table 2:** LAMP primer set used in this study (in bold) and their properties.

Label	Length	Tm	5’dG	3’dG	GC Rate	Sequence
**F3**	21	55.33	−4.9	−4.18	0.38	**ACTGACTTAACAAAGCCTTAC**
**B3**	20	56.19	−3.53	−4.55	0.4	**ACTAGTGGTCCAAAACTTGT**
**FIP**	50					**GGTGGTATGTCTGATCCCAATATTT-GGGATTTGTTAAAATATGACTTCAC**
**BIP**	45					**TTGTGTTAACTGTTTGGATGACAGA-AGTGGGAACACTGTAGAGAA**
F2	25	56.51	−4.85	−4.51	0.32	GGGATTTGTTAAAATATGACTTCAC
F1c	25	60.56	−6	−2.28	0.4	GGTGGTATGTCTGATCCCAATATTT
B2	20	57.27	−5.84	−4.1	0.45	AGTGGGAACACTGTAGAGAA
B1c	25	60.88	−4.72	−4.76	0.36	TTGTGTTAACTGTTTGGATGACAGA
**LF**	25	60.92	−6.19	−4.1	0.4	**CGGTCAAAGAGTTTTAACCTCTCTT**
**LB**	21	60.35	−4.96	−5.17	0.43	**TGCATTCTGCATTGTGCAAAC**

## Data Availability

Not applicable.
